# The pseudokinase NRBP1 activates Rac1/Cdc42 via P-Rex1 to drive oncogenic signalling in triple-negative breast cancer

**DOI:** 10.1038/s41388-023-02594-w

**Published:** 2023-01-24

**Authors:** Xue Yang, Miguel I. Cruz, Elizabeth V. Nguyen, Cheng Huang, Ralf B. Schittenhelm, Jennii Luu, Karla J. Cowley, Sung-Young Shin, Lan K. Nguyen, Terry C. C. Lim Kam Sian, Kimberley C. Clark, Kaylene J. Simpson, Xiuquan Ma, Roger J. Daly

**Affiliations:** 1 Cancer Program, Biomedicine Discovery Institute, Monash University, Melbourne, VIC 3800 Australia; 2 Department of Biochemistry and Molecular Biology, Monash University, Melbourne, VIC 3800 Australia; 3 Monash Proteomics and Metabolomics Facility, Monash University, Melbourne, VIC 3800 Australia; 4 Victorian Centre for Functional Genomics, Peter MacCallum Cancer Centre, Melbourne, VIC Australia; 5 Sir Peter MacCallum Department of Oncology, University of Melbourne, Parkville, VIC Australia

**Keywords:** Breast cancer, Cell signalling

## Abstract

We have determined that expression of the pseudokinase NRBP1 positively associates with poor prognosis in triple negative breast cancer (TNBC) and is required for efficient migration, invasion and proliferation of TNBC cells in culture as well as growth of TNBC orthotopic xenografts and experimental metastasis. Application of BioID/MS profiling identified P-Rex1, a known guanine nucleotide exchange factor for Rac1, as a NRBP1 binding partner. Importantly, NRBP1 overexpression enhanced levels of GTP-bound Rac1 and Cdc42 in a P-Rex1-dependent manner, while NRBP1 knockdown reduced their activation. In addition, NRBP1 associated with P-Rex1, Rac1 and Cdc42, suggesting a scaffolding function for this pseudokinase. NRBP1-mediated promotion of cell migration and invasion was P-Rex1-dependent, while constitutively-active Rac1 rescued the effect of NRBP1 knockdown on cell proliferation and invasion. Generation of reactive oxygen species via a NRBP1/P-Rex1 pathway was implicated in these oncogenic roles of NRBP1. Overall, these findings define a new function for NRBP1 and a novel oncogenic signalling pathway in TNBC that may be amenable to therapeutic intervention.

## Introduction

Triple negative breast cancer (TNBC), lacking the expression of ER, PR and HER2, is an aggressive subtype of breast cancer characterized by poorer prognosis, higher tumour grade and greater tumour burden [1]. Despite the recent introduction of specific immunotherapies and PARP inhibitors for treatment of certain subpopulations of TNBC patients, there remains a paucity of targeted treatments for TNBC and cytotoxic chemotherapy is still the cornerstone of treatment [2]. Consequently, there is an urgent need to identify novel targeted and personalized treatment strategies for patients suffering from TNBC.

Approximately 10% of annotated human protein kinases are classified as pseudokinases, since they lack at least one of the conserved amino acid motifs DFG, HRD and VAIK that are critical for catalytic function [3, 4]. In the absence of catalytic activity, pseudokinases can modulate cellular signalling by functioning as scaffolds, anchors or allosteric regulators [4, 5]. Nuclear receptor binding protein 1 (NRBP1) is a multidomain pseudokinase that is highly conserved from worms to humans and ubiquitously expressed [6]. Structurally, NRBP1 contains two nuclear receptor-binding motifs, a glutamate- and serine-rich region, a kinase-like domain, nuclear export and localization signals, a myeloid leukaemia factor 1 (MLF1)-binding region, an Elongin BC-binding box and a transforming growth factor β1-stimulated clone 22 (TSC22)-binding region [6]. A variety of binding partners have been described for NRBP1, indicating that it may regulate diverse processes. For example, NRBP1 functions as a substrate recognition factor of a Cullin RING ubiquitin ligase (CRL) complex [7, 8], and also binds to particular transcription factors including TSC22D2, TSC22D4 and Sall4 [7]. Other binding partners include the MLF1 oncoprotein [9], the small G protein Rac3 [10] and Jab1, a member of the COP9 signalosome complex and AP1 regulator [11].

Over the last decade, strong evidence has emerged that NRBP1 plays context-specific roles in a variety of cancers, including colorectal, lung, prostate, bladder and breast cancers [7, 12–16]. A tumour suppressor role for NRBP1 was first highlighted by a genetic screen in *C. elegans* and gene knock-out studies in mice, with the latter reporting haematological and intestinal tumours [7]. Consistent with these data, high NRBP1 expression in colorectal cancer (CRC) correlates with better prognosis, and overexpression of NRBP1 in CRC cell lines triggered cell apoptosis and inhibited cell proliferation in vitro, and reduced xenograft growth in vivo [17]. Similarly, in lung adenocarcinoma, high NRBP1 expression is associated with good prognosis [7]. However, in other cancers, NRBP1 appears to play an oncogenic role, although the detailed molecular mechanisms are lacking. For example, in prostate and bladder cancer, NRBP1 expression is positively associated with poor clinical outcomes, and silencing of NRBP1 leads to decreased proliferation, and for the latter cancer, reduced xenograft growth [14, 16]. In breast cancer, its role remains controversial. One study reported that NRBP1 negatively regulates cell proliferation in two breast cancer cell lines [15]. However, this is contradicted by data from comprehensive functional genomic screens across cancer cell lines identifying NRBP1 as a context-specific fitness gene, specifically in breast cancer [12].

In this study, we identified NRBP1 via a proteomics screen in TNBC and then characterized its function and signalling mechanism in detail. Utilizing diverse functional assays, both in vitro and in vivo, coupled with a BioID/MS screen, we demonstrate that NRBP1 plays a positive role in TNBC growth and metastasis and determine that this is mediated via a novel pathway involving NRBP1 and the Rac1 GEF P-Rex1, itself a known breast cancer oncogene [18].

## Results

### Identification of NRBP1 as a pseudokinase implicated in triple-negative breast cancer

**Fig. 1:**
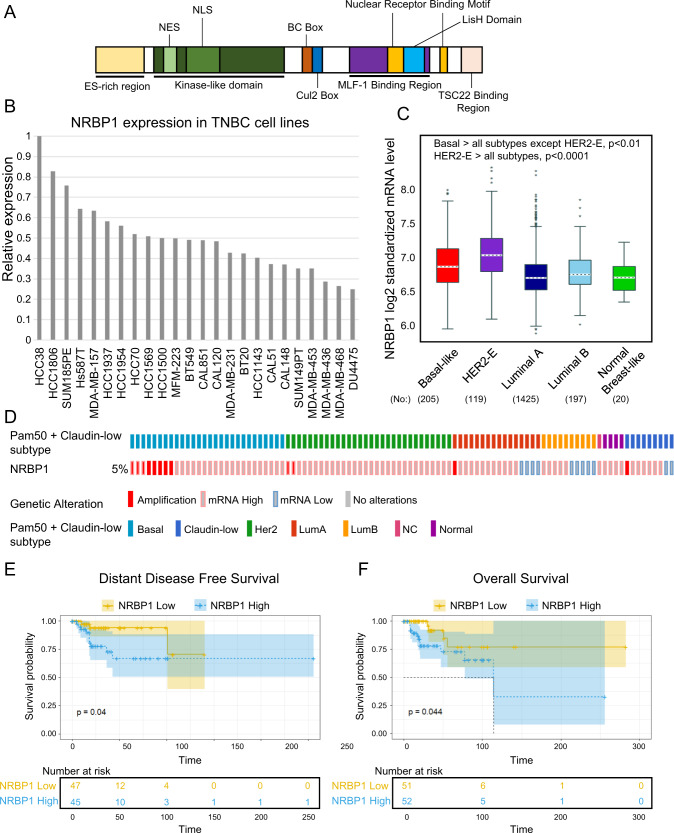
NRBP1 and its expression in breast cancer. **A** Schematic representation of NRBP1 structure. Abbreviations: ES-rich, glutamate- and serine-rich, NES nuclear export signal, NLS nuclear localization signal, BC Box elongin B and C binding box. **B** Expression of NRBP1 protein across a panel of 24 TNBC cell lines. This was determined by mass-spectrometry-based proteomics as described in Materials and methods**. C** Expression of NRBP1 mRNA across PAM50 molecular subtypes. **D** NRBP1 gene alteration and mRNA expression across PAM50 and claudin-low breast cancer subtypes. Only 5 % of breast cancers with NRBP1 gene alterations are shown for ease of visualization. **E–F** Relationship between NRBP1 expression and TNBC patient prognosis. Graphs showing relationship with distant disease-free survival (**E**) and overall survival (**F**) are shown.

In order to identify protein kinases that may regulate progression of TNBC and represent potential therapeutic targets, we undertook mass spectrometry (MS)-based proteomic profiling across a panel of 24 TNBC cell lines [19, 20] to identify protein kinases with increased expression/activation in particular cell line subsets. This led to the identification of the multidomain pseudokinase NRBP1 (Fig. 1A) on the basis that it exhibited marked variation in protein expression across the panel (Fig. 1B). Interrogation of publicly-available data revealed that NRBP1 gene expression was significantly higher in the Basal Subgroup (predominantly TNBC) and HER2 cancers, compared to the other PAM50 subgroups (Fig. 1C). In addition, while NRBP1 gene amplification only occurred with low frequency, this was detected in TNBCs (Fig. 1D). High NRBP1 expression positively correlated with poor distant disease-free and overall survival of TNBC patients (Fig. 1E, F). These data indicated a potential role for NRBP1 in promoting TNBC progression and highlighted this protein for further functional and mechanistic interrogation.

### Dependency of TNBC cell lines on NRBP1 for efficient cell migration, invasion and proliferation

**Fig. 2:**
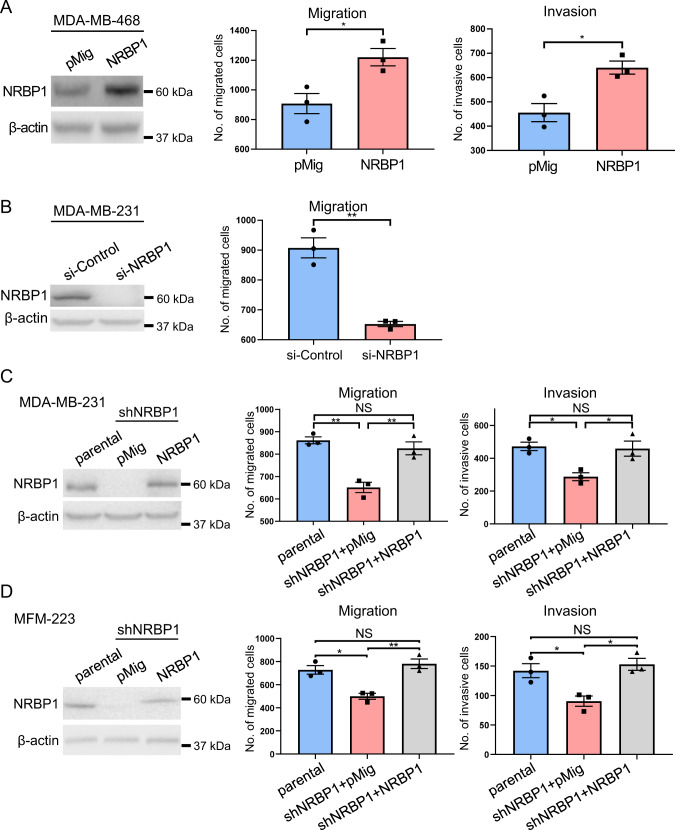
NRBP1 positively regulates TNBC cell migration and invasion. **A** Overexpression of NRBP1 in MDA-MB-468 cells enhances cell migration and invasion. Left panel, Western blots of cell lysates from cells transfected with an NRBP1 expression vector or empty vector (pMig). Graphs on right, results from transwell assays. Representative images are shown in Supplementary Fig. 1A. **B** Knockdown of NRBP1 reduces cell migration in MDA-MB-231 cells. NRBP1 was knocked down using a siRNA pool, with successful knockdown confirmed by Western blotting (left panel). Graph on right, results from transwell assays. Results for deconvolution of the siRNA pool are shown in Supplementary Fig. 1B. Stable knockdown of NRBP1 in TNBC cells using shRNA results in reduced migration and invasion. Results shown are for MDA-MB-231 (**C**) and MFM-223 cells (**D**). Cells were subjected to shRNA-mediated NRBP1 knockdown and then rescue with either empty vector or a shRNA-resistant NRBP1 construct. MDA-MB-231 parental (**C**) and MFM-223 parental cells (**D**) were also included as controls. Western blotting data and results for transwell migration and invasion assays are shown. Note that the exogenous NRBP1 has a slightly decreased mobility due to the presence of the Flag-tag. Error bars represent the standard error of the mean from *n* = 3 independent assays. NS indicates *p* > 0.05, *p < 0.05, **p < 0.01 by student’s t-test (**A**, **B**) or two-way ANOVA with Tukey’s multiple comparisons test (**C**, **D**).

Next, we undertook functional characterization of NRBP1 in cell culture models of TNBC using a variety of biological assays. Overexpression of NRBP1 in MDA-MB-468 cells, which exhibit relatively low endogenous expression of the pseudokinase (Fig. 1B), significantly enhanced cell migration and invasion (Fig. 2A, Supplementary Fig. 1A). In contrast, siRNA-mediated knockdown of NRBP1 led to significantly decreased migration and invasion of MDA-MB-231 cells (Fig. 2B, Supplementary Fig. 1B, C), and reduced migration of CAL-120 and MFM-223 cells (Supplementary Fig. 1D). To validate the role of NRBP1 further, we knocked down NRBP1 by stably expressing a Dox-inducible shRNA (shNRBP1#1) in MDA-MB-231 and MFM-223 TNBC cells. This resulted in significantly reduced cell migration and invasion that could be rescued via expression of a shRNA-resistant NRBP1 construct (Fig. 2C, D).

**Fig. 3:**
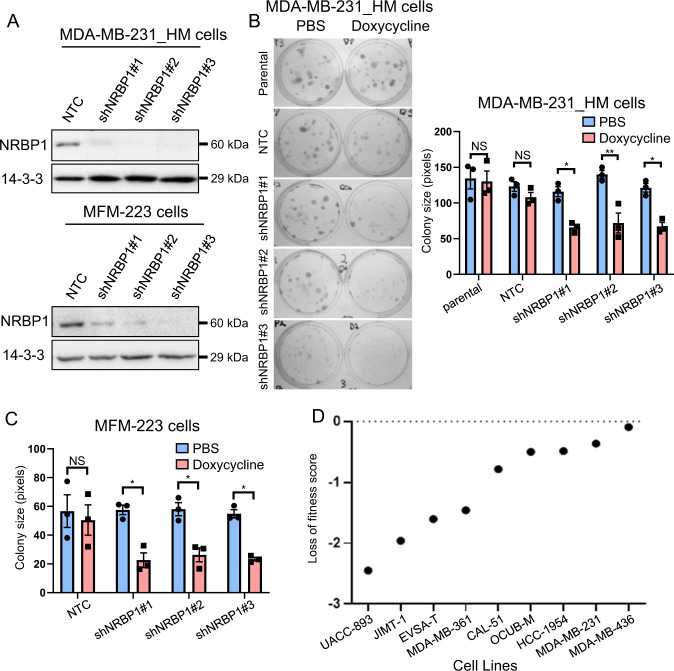
NRBP1 knockdown inhibits TNBC cell growth. **A** Confirmation of inducible knockdown. Western blotting of lysates from MDA-MB-231_HM cells (top panel) and MFM-223 cells (bottom panel) expressing different doxycycline-inducible NRBP1 shRNAs. Cells were treated with doxycycline or PBS control for 48 h (MDA-MB-231_HM cells) or 72 h (MFM-223 cells) prior to lysis. **B** NRBP1 knockdown reduces colony formation by MDA-MB-231_HM cells. Left panel, images of plates at Day 11. Right panel, quantification of colony size. **C** NRBP1 knockdown reduces colony formation by MFM-223 cells. Graph shows quantification of colony size at Day 23. Representative images are shown in Supplementary Fig. 2B. Error bars represent the standard error of the mean from *n* = 3 independent experiments. NS indicates *p* > 0.05, *p < 0.05, **p < 0.01 by one-way ANOVA with Tukey’s multiple comparisons test. **D** NRBP1 represents a breast cancer context-dependent fitness gene. Results for CRISPR-Cas9 screens across different breast cancer cell lines, extracted from the DepMap portal. A loss of fitness score < 0 indicates a statistically significant decrease in proliferation after NRBP1 knockdown in comparison with controls and therefore increased dependency of the cell line for NRBP1 [12].

In order to extend our analyses to cell proliferation and also mouse models, we expressed three different Dox-inducible NRBP1 shRNAs (shNRBP1#1, shNRBP1#2 and shNRBP1#3) in the MDA-MB-231_HM cell line, a highly metastatic variant [21], as well as MFM-223 cells. NRBP1 knockdown efficiency after doxycycline induction was validated by Western blotting (Fig. 3A). Following NRBP1 knockdown, MDA-MB-231_HM cell proliferation in monolayer was significantly decreased (Supplementary Fig. 2A). In addition, colony formation ability was impaired in both MDA-MB-231_HM and MFM-223 cells upon NRBP1 knockdown (Fig. 3B, C, Supplementary Fig. 2B). Further supporting a pro-proliferative role for NRBP1 in breast cancer, data extracted from the Dependency Map (DepMap) portal indicated that NRBP1 is a breast cancer context-specific fitness gene required for the efficient proliferation of multiple breast cancer cell lines, including the TNBC lines MDA-MB-231, CAL51, HCC-1954 and MDA-MB-436 (Fig. 3D) [12]. Of note, knockdown of NRBP1 in either MDA-MB-231 or MFM-223 cells did not induce apoptosis, as determined by Western blotting for cleaved PARP (Supplementary Fig. 2C). Overall, these data indicated that NRBP1 is required for efficient TNBC cell migration, invasion and proliferation in vitro.

### Determination of the role of NRBP1 in TNBC growth and metastasis

**Fig. 4:**
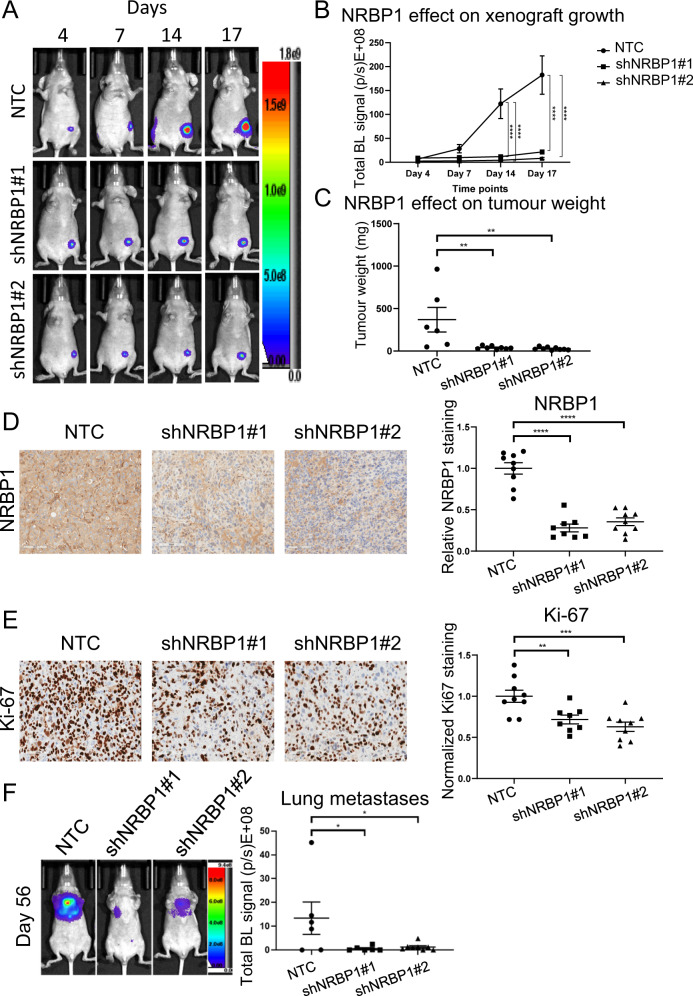
NRBP1 is required for efficient TNBC xenograft growth and experimental metastasis. **A** NRBP1 knockdown reduces growth of MDA-MB-231_HM orthotopic xenografts. Representative whole body BLI images from each group at different time points are shown. NTC, non-targeting control. **B** Quantification of xenograft growth. Mean tumour luciferase intensities over time were measured as mean photon counts per sec. Each group exhibited *n* ≥ 8 mice. **C** Quantification of tumour weight. Data at Day 34 post-injection were obtained from NTC (*n* = 6), shNRBP1#1 (*n* = 8) and shNRBP1#2 (*n* = 9) mice. Three mice from the original NTC group reached ethical endpoint earlier at Day 27 and were excluded from the tumour weight analysis. However, these mice were used for IHC analysis (below). **D** Confirmation of NRBP1 knockdown by IHC. **E** NRBP1 knockdown reduces tumour cell proliferation. Representative IHC staining for Ki67 is shown. For **D**, **E**, data were quantified from NTC (*n* = 9), shNRBP1#1 (*n* = 8) and shNRBP1#2 (*n* = 9) mice. All xenografts were collected at the endpoint of experiment (at Day 34), except for three xenografts from control mice that were collected at Day 27 when tumour reached ethical endpoint. **F** NRBP1 is required for efficient TNBC metastasis. Left panel, representative whole body BLI images from each group at Day 56. Right panel, luciferase intensities of tumour metastasis to lung were generated from NTC (*n* = 6), shNRBP1#1 (*n* = 6) and shNRBP1#2 (*n* = 8) mice. For **B**–**F**, error bars represent the standard error of the mean. *p < 0.05, **p < 0.01, ***p < 0.001, ****p < 0.0001 by two-way ANOVA with Dunnett’s multiple comparison test (**B**), or one-way ANOVA with Dunnett’s multiple comparisons test (**C**–**F**).

To evaluate the role of NRBP1 in TNBC growth in vivo, MDA-MB-231_HM cells stably expressing luciferase and one of two different Dox-inducible NRBP1 shRNAs were injected into the mammary fat pad of BALB/c athymic nude mice and xenograft growth was monitored via non-invasive bioluminescence imaging. Compared to vector control, suppression of NRBP1 by each shRNA markedly reduced xenograft growth (Fig. 4A–C), with efficient NRBP1 knockdown confirmed by immunohistochemical staining (Fig. 4D). In addition, NRBP1 knockdown was associated with reduced Ki67 staining of tumour sections, indicating that this leads to lower MDA-MB-231_HM cell proliferation in vivo (Fig. 4E). To assess the impact of NRBP1 on TNBC metastasis, an experimental metastasis model was utilized, where MDA-MB-231_HM cells expressing Dox-inducible NRBP1 shRNA were injected into the tail vein of BALB/c athymic nude mice. The results indicated that compared to vector control, NRBP1 knockdown by either shRNA significantly reduced tumour metastasis to lung (Fig. 4F). These in vivo data lend further support to an oncogenic role for NRBP1 in TNBC.

### Characterization of the NRBP1 interactome in TNBC

**Fig. 5:**
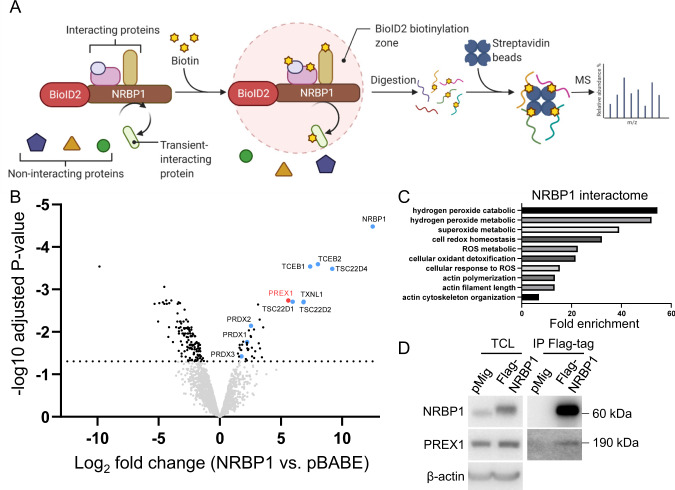
Characterization of the NRBP1 interactome by BioID-MS. **A** Schematic workflow of BioID-MS. A promiscuous biotin ligase (BirA*) is fused to the protein of interest (the ‘bait’ protein, here NRBP1) and the fusion protein expressed in cells. Addition of excess biotin results in biotinylation of endogenous proteins in the proximity of the bait. After affinity pulldown using streptavidin agarose beads, the biotinylated proteins are identified by MS. The figure was adapted from “BioID Assay”, using BioRender.com (2022). **B** The NRBP1 interactome in TNBC. A volcano plot representation of the MS dataset with cut-off of adjusted *p*-value ≤ 0.05 and Log2 fold change ≥ 1. Data are generated from *n* = 3 independent experiments. Labelled proteins represent previously-identified interactors or novel ones associated with cytoskeletal or redox regulation. **C** Functional pathways associated with NRBP1 interactors. The graph shows the top ten functions of interactors identified by “Metascape” software. **D** Confirmation of NRBP1-P-Rex1 interaction. Flag-tagged NRBP1 or vector control were transfected into MFM-223 cells. Total cell lysate (TCL) and Flag IPs were subjected to Western blotting as indicated.

While MS has been previously used to identify NRBP1 binding partners [7], the context-specific roles of NRBP1 in cancer indicate that the NRBP1 interactome may vary according to cell type. Consequently, we sought to define the interactome in TNBC using the BioID-MS method (Fig. 5A). MDA-MB-231_EcoR cells stably expressing mycBioID2-NRBP1 were established and treated with biotin prior to lysate preparation and affinity pulldown using streptavidin-coupled agarose beads. Bound proteins were then analysed by MS (Fig. 5A). A total of 41 proteins, including NRBP1 itself, were significantly enriched in mycBioID2-NRBP1 samples compared to the vector control (Fig. 5B, Supp Table 1), indicating that NRBP1 is either proximal to or interacts with these proteins. Amongst these proteins, TSC22D1, TSC22D2, TSC22D4, TCEB1 (Elongin B) and TCEB2 (Elongin C) are previously identified interactors of NRBP1 [7], while the Rac1 GEF P-Rex1, together with Thioredoxin-like 1 (TXNL1) and Peroxiredoxin (PRD)1-3 represented novel top-ranked candidates. Upon pathway analysis of NRBP1 interactors, ROS-related and cytoskeleton-regulated processes were identified as the top ten enriched functional categories (Fig. 5C), indicating that they are important pathways involved in NRBP1-regulated cellular events. Since P-Rex1 is a known regulator of cytoskeletal organization and cell migration, positively regulates ROS production and promotes breast cancer development and metastasis [18, 22, 23], we selected this candidate for further characterization. To confirm the interaction between NRBP1 and P-Rex1, immunoprecipitation (IP)/Western blotting analyses were performed in MFM-223 cells expressing Flag-tagged NRBP1. Consistent with the MS data, endogenous P-Rex1 could be detected by Western blotting of Flag-NRBP1 IPs (Fig. 5D).

Interrogation of P-Rex1 expression across a large panel of breast cancer cell lines from the CCLE revealed that P-Rex1 expression was significantly higher in luminal than basal/TNBC cell lines (Supplementary Fig. 3A), consistent with characterization of P-Rex1 expression in primary breast cancers [18, 24]. However, P-Rex1 expression was clearly detectable in the majority of the TNBC cell lines, confirming P-Rex1 as a potential mediator of NRBP1 signalling in TNBC. Of note, while one paper reported undetectable levels of P-Rex1 in MDA-MB-231 cells [25], interrogation of the CCLE reveals low P-Rex1 mRNA expression levels in these cells (Supplementary Fig. 3B), consistent with a previous paper [24].

### P-Rex1/Rac1/Cdc42 represents a novel NRBP1 signalling axis

**Fig. 6:**
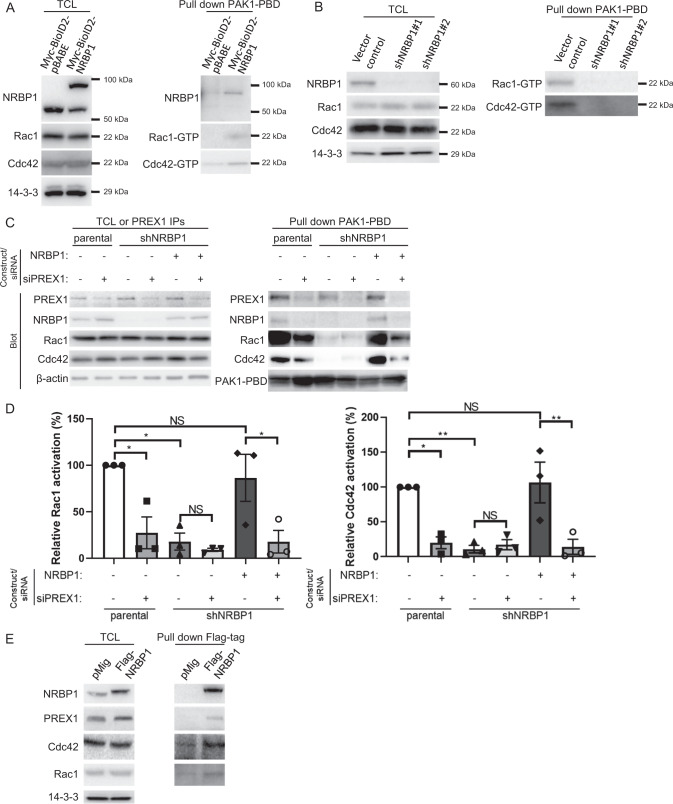
NRBP1 regulates Rac1/Cdc42 activation in TNBC via P-Rex1. **A** NRBP1 overexpression enhances activation of Rac1 and Cdc42. Results of PAK-1 PBD pull-down assays for active Rac1/Cdc42 using MDA-MB-231_EcoR cells expressing mycBioID2-NRBP1 or the empty vector. Cell lysates and PAK-1 PBD pulldown assays were Western blotted as indicated. Densitometry results of Rac1/Cdc42 activation are shown in Supplementary Fig. 4A. Data are representative of *n* = 3 independent experiments. **B** Stable knockdown of NRBP1 reduces activation of Rac1 and Cdc42. Vector control and NRBP1 knockdown MDA-MB-231_HM cells were treated with doxycycline prior to lysis. Cell lysates and PAK-1 PBD pulldown assays were subjected to Western blotting as indicated. Densitometry results of Rac1/Cdc42 activation are shown in Supplementary Fig. 4B. Data are representative of *n* = 3 independent experiments. **C**, **D** NRBP1-mediated activation of Rac1 and Cdc42 is P-Rex1-dependent. MDA-MB-231-shNRBP1 cells were complemented with shRNA-resistant NRBP1 in the presence or absence of siRNA-mediated P-Rex1 knockdown. Parental cells+/− P-Rex1 knockdown were also included as controls. Cell lysates and PAK-1 PBD pulldown assays were then Western blotted as indicated. Confirmation of P-Rex1 knockdown was obtained by Western blotting of P-Rex1 IPs. Representative blots are shown in (**C**), and densitometry results of Rac1/Cdc42 activation are shown in (**D**). The PAK1-PBD band is obtained as a cross-reacting signal when blotting with the Rac1 antibody and serves as a loading control for the PAK1- PBD protein. Data are representative of *n* = 3 independent experiments. Rac1/Cdc42 activation was normalized to total Rac1/Cdc42, which was normalized to β-actin. Data are expressed relative to parental cells with si-Control, which was arbitrarily set at 100%. Error bars represent the standard error of the mean from *n* = 3 independent experiments. NS indicates *p* > 0.05, *p < 0.05, **p < 0.01 by two-way ANOVA with Tukey’s multiple comparisons test. **E** Association of NRBP1 with P-Rex1, Rac1 and Cdc42. MFM-223 cells were transfected with Flag-tagged NRBP1. Anti-Flag IPs were then Western blotted as indicated.

P-Rex1 is best characterized as a GEF activating Rac1, although positive regulation of Cdc42 by P-Rex1 has also been reported [26–28]. Both Rac1 and Cdc42 are Rho family GTPases that are key players in cell growth, migration, invasion and metastasis [27]. Consequently, key questions were how does NRBP1 impact Rac1/Cdc42 activity, and whether any biological effects of NRBP1 are P-Rex1-dependent. To address the first question, pulldown of GTP-bound Rac1/Cdc42 was performed using lysates from MDA-MB-231_EcoR cells expressing mycBioID2-NRBP1, revealing that NRBP1 overexpression increased the activation levels of Rac1 and Cdc42 (Fig. 6A, Supplementary Fig. 4A). Interestingly, NRBP1 was detectable in pulldowns from the overexpressing cells, indicating that NRBP1 associates with the complex of PAK1-PBD with active Rac1 or Cdc42. Also, the pulldown assay was performed using lysates from MDA-MB-231_HM cells exhibiting shRNA-mediated NRBP1 knockdown. Here, ablation of NRBP1 expression markedly reduced levels of activated Rac1 and Cdc42 (Fig. 6B, Supplementary Fig. 4B). These results indicated that NRBP1 is required for, and can enhance, the activation of Rac1/Cdc42 in TNBC cells.

Next, we determined the requirement for P-Rex1 in NRBP1-mediated Rac1/Cdc42 activation. In MDA-MB-231 cells with shRNA-mediated NRBP1 knockdown, expression of shRNA-resistant NRBP1 rescued activation of Rac1 and Cdc42, but this effect was blocked upon concomitant P-Rex1 knockdown (Fig. 6C, D). A similar result was obtained in MFM-223 cells, with rescue by shRNA-resistant NRBP1 being P-Rex1-dependent for Rac1, and a strong trend for P-Rex1 dependency for Cdc42 (Supplementary Fig. 4C, D). Two additional points are worthy of mention in the context of these experiments. First, NRBP1 knockdown did not affect P-Rex1 expression. Second, in addition to NRBP1 and activated Rac1/Cdc42, P-Rex1 was also detected in the PAK1-PBD pulldowns, suggesting formation of a higher-order complex involving these proteins. In order to determine whether complex formation could be detected without the use of PAK1-PBD as an affinity reagent, Western blotting was undertaken on IPs of Flag-tagged NRBP1 expressed in MFM-223 cells. Indeed, endogenous Rac1, Cdc42 and P-Rex1 could be detected in these IPs (Fig. 6E). Overall, these data reveal a novel NRBP1/P-Rex1/Rac1/Cdc42 signalling axis in TNBC, that may involve a scaffolding function of NRBP1.

### NRBP1 promotes growth, migration and invasion of TNBCs through the P-Rex1/Rac1/Cdc42 signalling axis

**Fig. 7:**
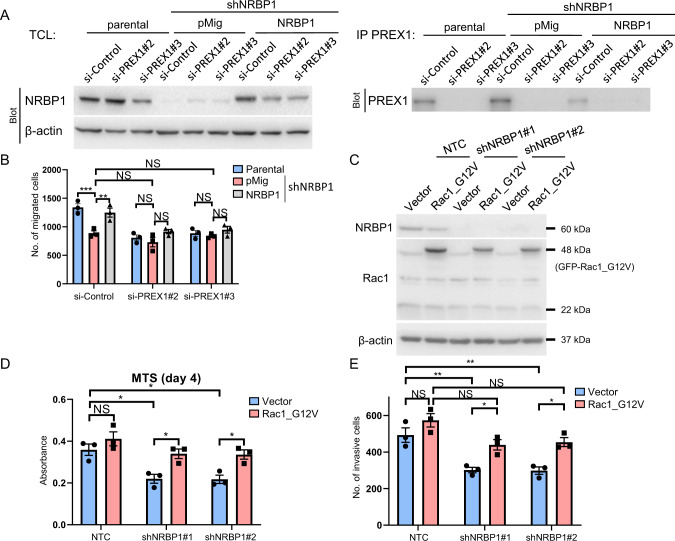
The P-Rex1/Rac1 axis plays essential roles in cellular functions regulated by NRBP1. **A** Western blotting validation of NRBP1 knockdown, NRBP1 rescue and P-Rex1 knockdown. MDA-MB-231 cells with shRNA-mediated NRBP1 knockdown were complemented with expression of shRNA-resistant NRBP1 in the presence or absence of P-Rex1 knockdown. Parental cells+/− P-Rex1 knockdown were also included as controls. Total cell lysates or P-Rex1 IPs were Western blotted as indicated. **B** Rescue of cell migration by NRBP1 is P-Rex1 dependent. Cells validated in (**A**) were subjected to transwell assays. **C** Expression of Rac1_G12V. MDA-MB-231 cells with shRNA-mediated NRBP1 knockdown were programmed to express GFP-Rac1_G12V. Cell lysates were Western blotted as indicated. **D** Effect of active Rac1 on monolayer proliferation of NRBP1-depleted cells. Cells validated in (**C**) were subjected to MTS assays. Data were obtained at Day 4. **E** Effect of active Rac1 on invasion of NRBP1-depleted cells. Cells validated in (**C**) were subjected to transwell assays. For **B**, **D** and **E**, error bars represent the standard error of the mean from *n* = 3 independent experiments. NS indicates *p* > 0.05, *p < 0.05, **p < 0.01, ***p < 0.001 by two-way ANOVA with Tukey’s multiple comparisons test.

To further interrogate the role of P-Rex1 in NRBP1 signalling, we determined the requirement for P-Rex1 in NRBP1-mediated biological effects. Expression of shRNA-resistant NRBP1 in MDA-MB-231 cells with knockdown of endogenous NRBP1 rescued the decreased cell migration, but this effect was P-Rex1 dependent, as demonstrated by siRNA-mediated knockdown of P-Rex1 (Fig. 7A-B). Similarly, shRNA-resistant NRBP1 was unable to restore cell invasion in knockdown cells in the absence of this GEF (Supplementary Fig. 5A). These data complement the signalling assays in Fig. 6C, D and Supplementary Fig. 4C, D and demonstrate that NRBP1 requires P-Rex1 for key functional effects in TNBC cells.

We also characterized the role of Rac1 in NRBP1 downstream signalling, based on the premise that active Rac1 should at least partially rescue the biological effects of NRBP1 knockdown. Here, the constitutively active Rac1_G12V mutant was stably expressed in either control or NRBP1 knockdown MDA-MB-231 cells (Fig. 7C). While this had no effect on monolayer proliferation of the control cells, expression of active Rac1 rescued the decreased cell proliferation observed upon NRBP1 knockdown (Fig. 7D). Similarly, rescue was also observed in cell invasion assays (Fig. 7E). In the case of cell migration, Rac1_G12V enhanced this endpoint in the control cells and also rescued migration in the knockdown cells, which is more consistent with a dominant positive effect (Supplementary Fig. 5B). Interestingly, expression of the active Cdc42 mutant Q61L also rescued cell proliferation and invasion of NRBP1-knockdown cells (Supplementary Fig. 5C–E), indicating that both active Rac1 and Cdc42 can compensate for NRBP1 deficiency, at least in terms of these two biological endpoints.

### The NRBP1 signalling axis in TNBC regulates reactive oxygen species

The identification of a novel pathway linking NRBP1 to P-Rex1 and Rac1/Cdc42 raised the important question of what downstream effectors might be engaged by NRBP1 signalling. To address this, we first assayed known downstream effectors of Rac1/Cdc42 by Western blot. However, overexpression of NRBP1 in MDA-MB-468 cells did not significantly affect total or relative (normalized for total protein) levels of phosphorylated PAK, LIMK or cofilin (Supplementary Fig. 6A–D). Similarly, changes in phosphorylation of LIMK or cofilin were not observed upon knockdown of NRBP1 in MDA-MB-231 cells and then rescue with shRNA-resistant NRBP1 (phosphorylated PAK could not be reproducibly detected in these cells) (Supplementary Fig. 7A–C). To further interrogate signalling events downstream of NRBP1, we compared control and NRBP1-knockdown cells by MS-based proteomic profiling. In the proteomic dataset, three out of the top 10 functional categories significantly enriched amongst differentially expressed proteins were cell cycle-related (Supp Tables 2, 3), consistent with the effect of NRBP1 on cell proliferation.

**Fig. 8:**
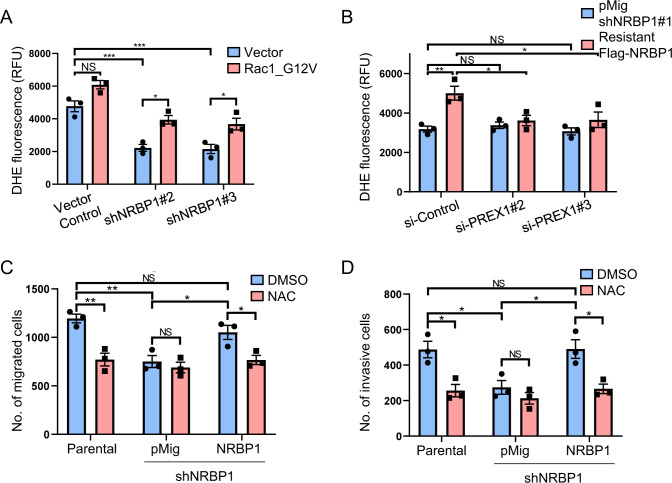
The NRBP1 signalling axis in TNBC regulates reactive oxygen species. **A** Roles of NRBP1 and Rac1 in regulating ROS in MDA-MB-231 cells. Cells with stable NRBP1 knockdown were programmed to express GFP-Rac1_G12V and cellular ROS was assayed. Data are quantified using relative fluorescence units (RFU). **B** Rescue of ROS by NRBP1 complementation is P-Rex1 dependent. MDA-MB-231 cells with shRNA-mediated NRBP1 knockdown were complemented by expression of shRNA-resistant NRBP1 in the presence or absence of P-Rex1 knockdown, and cellular ROS was assayed. Data are quantified using relative fluorescence units (RFU). **C**, **D** NRBP1-promoted cell migration and invasion requires ROS. MDA-MB-231 cells with shRNA-mediated NRBP1 knockdown were complemented by expression of shRNA-resistant NRBP1 in the presence or absence of NAC. Parental cells+/− NAC were also included as a control. Data shown are from transwell migration (**C**) and invasion (**D**) assays. Error bars represent the standard error of the mean from *n* = 3 independent experiments. NS indicates *p* > 0.05, *p < 0.05, **p < 0.01, ***p < 0.001 by two-way ANOVA with Tukey’s multiple comparisons test.

Since reactive oxygen species (ROS)-related processes represented major enriched functional categories for NRBP1 interactors identified by BioID/MS (Fig. 5C), and P-Rex1 and Rac1/Cdc42 are known to promote ROS generation in a context-specific manner [29–31], we also characterized the role of ROS in NRBP1-regulated biological endpoints. Knockdown of NRBP1 using shRNAs led to a significant decrease in ROS levels, and this was rescued by expression of constitutively active Rac1 or Cdc42 (Fig. 8A and Supplementary Fig. 8). Furthermore, depletion of P-Rex1 blocked the ability of shRNA-resistant NRBP1 to rescue ROS levels in NRBP1-depleted cells (Fig. 8B). To determine whether the functional roles of NRBP1 are dependent on ROS, N-acetylcysteine (NAC), a commonly used small molecule that removes ROS, was applied. Notably, treatment with NAC significantly inhibited migration and invasion of control cells but did not affect NRBP1 knockdown cells. Moreover, it significantly inhibited the ability of shRNA-resistant NRBP1 to rescue these biological endpoints (Fig. 8C, D). Overall, these data highlight ROS generation as a key downstream signalling mechanism of the NRBP1/P-Rex1 axis.

## Discussion

In this manuscript we present strong evidence from both in vitro and in vivo model systems as well as patient cohorts that the pseudokinase NRBP1 plays an oncogenic role in TNBC. This is consistent with its function in prostate and bladder cancer, but not CRC, where it appears to exhibit a tumour suppressor function [13, 14, 16]. In addition, we identify a novel signalling role for NRBP1 where it interacts with the GEF P-Rex1 and promotes Rac1/Cdc42 activation. This leads to a model where, like the pseudokinases KSR [32] and PEAK1-3 [33], NRBP1 functions as a scaffold to mediate assembly of oncogenic signalling complexes.

To date, the best-characterized role of NRBP1 is as substrate recognition factor of a CRL complex [7, 8], although so far, only BRI2 and BRI3, which regulate processing of the amyloid precursor protein, have been identified as targets of this complex [8]. However, while we identified Elongins B and C as NRBP1 interactors in TNBC cells, as well as specific TSC22D family members that can stabilize formation of a dimeric CRL complex [8], NRBP1 does not appear to target P-Rex1 for degradation, since P-Rex1 expression levels were not modulated by NRBP1, and NRBP1 positively regulated P-Rex1 signalling. Instead, the ability of NRBP1 to interact with P-Rex1 and activate Rac1/Cdc42 in a P-Rex1-dependent manner suggests that NRBP1 positively regulates P-Rex1 signalling by bringing P-Rex1 and Rac1/Cdc42 into close proximity to each other. This is supported by the presence of NRBP1, P-Rex1 and active Rac1/Cdc42 in PAK1-PBD pulldowns and of Rac1, Cdc42 and P-Rex1 in NRBP1 IPs. However, it remains possible that active Rac1/Cdc42 only associate with the NRBP1/P-Rex1 complex following activation, and this represents a mechanism for localizing Rac1/Cdc42 signalling. In this regard, it is noteworthy that activated Rac3 was previously identified as a NRBP1 binding partner that co-localized with NRBP1 at endomembranes and in lamellopodia [10]. Such a scaffolding function for NRBP1 would be similar to that characterized for ELMO1, which associates with both Rac1 and the Rac GEF DOCK5, and enhances the GEF activity of DOCK5 [34].

Of note, the original paper characterizing P-Rex1 reported that P-Rex1 activates Rac1 and not Cdc42, with these assays undertaken in insect Sf9 cells [35]. However, in that paper, P-Rex1 demonstrated GEF activity towards both Rac1 and Cdc42 in vitro, and a crystal structure has been reported for the P-Rex1 DH-PH domains bound to Cdc42, as well as to Rac1 [26]. Moreover, activation of Cdc42 by P-Rex1 has been described in mammalian HEK-293T cells [28]. Consequently, it appears that P-Rex1 exhibits context-dependent GEF activity towards Cdc42, and we propose that this is likely to be regulated by accessory proteins such as NRBP1 that may provide a scaffolding function. Supporting this hypothesis, analysis of the NRBP1 Bio-ID data did not reveal another GEF in addition to P-Rex1 that could be responsible for the activation of Cdc42, and knockdown experiments demonstrated the P-Rex1 dependency of NRBP1-mediated Cdc42 activation.

The identification of P-Rex1 as a NRBP1 binding partner sheds new light on the oncogenic role of NRBP1, not only due to the well-established roles of Rac1/Cdc42 in human cancer [27], but also because P-Rex1 has recently emerged as an important oncogene in its own right [23]. P-Rex1 is amplified or mutated in approximately 4% of human cancers [36] and overexpressed in many malignancies including melanoma and those of the breast, prostate, thyroid, kidney and ovary [23]. In breast cancer, high P-Rex1 expression is associated with the luminal A and B subtypes and associates with poor patient prognosis in the luminal B subtype [18, 24] and use of transgenic and gene knockout models demonstrate an important role for P-Rex1 in mammary tumour initiation and metastasis [18]. Consistent with these data, P-Rex1 is required for efficient metastasis in an NRas-driven mouse model of melanoma [37]. However, the higher expression of P-Rex1 in luminal versus TNBCs [18, 24] does not rule out this GEF from contributing to disease progression in TNBC. Indeed, we demonstrate that NRBP1 signals through P-Rex1 to promote cell migration and invasion in MDA-MB-231 TNBC cells, which express very low levels of this GEF. Instead, rather than P-Rex1 overexpression, we propose that it is increased expression of NRBP1 that is the critical factor. Given that NRBP1 and P-Rex1 are overexpressed in multiple, overlapping cancer types, it will be important to determine whether NRBP1 signals via P-Rex1 in malignancies other than breast cancer, their interdependency in cancers where they are both overexpressed, and how the combined expression of both proteins influences patient outcome.

A surprising result was that while NRBP1 positively regulated Rac1 and Cdc42 activity in a P-Rex1-dependent manner, it did not affect phosphorylation of PAK, LIMK and cofilin, downstream effectors of these GTPases that mediate regulation of the actin cytoskeleton [27]. However, it did positively impact ROS generation, and this pathway was critical for NRBP1-mediated effects on cell migration and invasion. The latter functional effects likely reflect known roles for ROS in regulating the actin cytoskeleton, including redox modification of actin and its regulators [29]. However, the mechanism underpinning the preferential effect of the NRBP1/P-Rex1 pathway on ROS versus PAK/LIMK is currently unclear. A possible mechanism is NRBP1-mediated localization of P-Rex1 to a specific subcellular compartment. It is also interesting to note that ROS-related pathways were significantly enriched in the NRBP1 interactome, reflecting the identification of not only P-Rex1 as a NRBP1 interactor but also the anti-oxidant proteins TXNL1 and PRDX1-3. This suggests that NRBP1 may regulate redox homeostasis by mechanisms beyond binding P-Rex1, which is known to signal via Rac to promote ROS generation [29].

The recruitment of multiple proteins by NRBP1 and its regulation of varied downstream pathways provides a likely explanation for the contrasting and context-specific effects of NRBP1 on tumour progression. For example, the ability of NRBP1 to function as a substrate recognition factor of a Cullin RING ubiquitin ligase (CRL) complex will depend on the expression profile of specific Cullins and substrates. In addition, the biological activity of particular pathways downstream of NRBP1 may be context-dependent, as exemplified by Cdc42 exhibiting oncogenic or tumour suppressor functions depending on cancer type [27]. A further mechanism that may be regulated in a context-specific manner is NRBP1 subcellular localization, since NRBP1 may shuttle between the cytoplasm and nucleus [6].

While pseudokinases lack protein kinase activity, they can still represent therapeutic targets. For example, if ATP binding is critical to pseudokinase function then the nucleotide binding site represents a potential target for small molecule drugs, and other possibilities include small molecule allosteric modulators and targeted degradative strategies [38]. In the case of NRBP1, further molecular characterization, including determination of the mechanism and function of the pseudokinase domain, is required to inform drug development programs. However, the identification of an oncogenic P-Rex1 signalling axis downstream of NRBP1 opens up possibilities in terms of therapeutic targeting. For example, small molecules that block P-Rex1 function have been identified [39]. In addition, the important role of ROS in NRBP1/P-Rex1 signalling raises the possibility of targeting antioxidant pathways, such as those mediated by NRF2/KEAP1, in order to raise ROS levels above those compatible with cell survival [40]. Consequently, this newly-identified NRBP1 signalling axis represents a potential target for precision treatment of TNBC, which urgently requires additional targeted therapeutic approaches.

## Materials and methods

### Plasmids

The cDNAs encoding N-terminal Flag-tagged NRBP1 purchased from Addgene (cat. 48197) and Flag-tagged shRNA-resistant NRBP1 synthesized by Genscript were cloned into the EcoRI restriction site of the pMig-GFP express vector. The cDNA encoding NRBP1 was cloned into the XhoI restriction site of the mycBioID2-pBABE-puro vector (Addgene, cat. 80900). The pMX-GFP-Rac1_G12V (cat. 14567) and pMX-GFP-Cdc42_Q61L plasmids (cat. 14568) were purchased from Addgene. The lentiviral expression vector pLKO-Tet-On containing either doxycycline-inducible non-targeting control (cat. VSC11653) or short hairpin RNA (shRNA) targeting NRBP1 (cat. V3SH11252) was purchased from Dharmacon RNAi Technologies (Horizon Discovery). NRBP1 shRNA catalogue numbers and sequences were #1: AATGAAGCCCAGCTGCACC, #2: TTCTCGACAAGTCTTCACA, #3: AATTGCTTCAGACTCCCAG.

### Antibodies and reagents

The following antibodies were purchased from Cell Signaling Technology: P-Rex1 (cat. 13168), Cdc42 (cat. 2462), Ki-67 (cat. 9027), Myc-tag (cat. 2276 S), PAK1/2/3 (cat. 2604), phospho-PAK1 (Thr423)/PAK2 (Thr402) (cat. 2601), LIMK1 (cat. 3842), LIMK2 (cat. 3845), phospho-LIMK1 (Thr508)/LIMK2 (Thr505) (cat. 3841), phospho-Cofilin (S3) (cat. 3313s) and cleaved PARP (cat. 9546s). Flag-tag (cat. F7425), P-Rex1 (cat. SAB2501302) and P-Rex1 (cat. HPA001927) were purchased from Sigma. β-actin (cat. sc-69879), pan 14-3-3 (cat. sc-1657), Cofilin (cat. sc-376476) and phospho-Cofilin (S3) (cat. sc-365882) were purchased from Santa Cruz Biotechnology. The following antibodies were also used: NRBP1 (Genetex, cat. GTX84007), Rac1 (EMD Millipore, cat. 05-389) and HRP-conjugated secondary antibodies against rabbit and mouse IgG (Bio-Rad, cat. 1706515, 1706516). Western blotting was undertaken as previously described [41].

N-acetylcysteine (NAC, cat. A7250) and mitomycin C (cat. M4287) were purchased from Sigma-Aldrich. Luciferin (Promega, cat. P1043) was used in animal work.

### Cell lines and tissue culture

HEK293T and Plat-E cells were maintained in Dulbecco’s Modified Eagle’s Medium (DMEM, Gibco, cat. 1200046) supplemented with 10% (v/v) fetal bovine serum (FBS; Fisher Biotech, catalogue no. S-FBS-US-015). TNBC cell lines were obtained from the American Type Culture Collection (ATCC) unless otherwise indicated. The MFM-223 line was obtained from Sigma Aldrich. MDA-MB-231 parental cells were obtained from EG&G Mason Research Institute, Worcester, MA. MDA-MB-231 cells stably expressing the murine ecotropic receptor (MDA-MB-231_EcoR) were a kind gift from A/Prof Maija Kohonen-Corish at the Garvan Institute of Medical Research. The highly metastatic MDA-MB-231 cell line (MDA-MB-231_HM) was originally established by Prof. Zhou Ou from Fudan University Shanghai Cancer Center in China. The MDA-MB-231_HM cell line expressing luciferase/mCherry was a kind gift from A/Prof. Erica Sloan at Monash University. In this paper, MDA-MB-231_HM refers to the cell line expressing luciferase/mCherry.

Breast cancer cell lines were cultured in RPMI-1640 medium (Life Technologies, catalogue no. 11875119 and Gibco, catalogue no. 31800-089) supplemented with 10% (v/v) FBS, 10 μg/mL Actrapid penfill insulin (Clifford Hallam Healthcare), and 20 mM HEPES (Life Technologies, catalogue no. 15630080). All the cell lines were authenticated by short tandem repeat polymorphism, single-nucleotide polymorphism and/or fingerprint analyses and underwent routine mycoplasma testing by PCR.

### Generation of stable cell lines

MDA-MB-231_EcoR cells stably expressing mycBioID2-pBabe-NRBP1 were generated by retroviral transduction [42]. To produce retroviral particles, Plat-E cells were transfected with mycBioID2-pBabe Express vectors using Lipofectamine 3000 (Life Technologies, cat. L3000015) in accordance with the manufacturer’s instructions. Cells were infected with retroviral particles for 24 h in the presence of polybrene (8 µg/ml; Millipore). Successfully transduced cells were selected using puromycin (1 µg/ml; Gibco, cat. A1113802) for 72 h.

For inducible knockdown of NRBP1, MDA-MB-231 parental, MDA-MB-231_HM and MFM-223 cells were infected with lentiviral particles generated by co-transfection of HEK-293T cells with VSVG, psPAX2 and shRNA-containing pLKO-Tet-On plasmids using Lipofectamine 3000. Successful transduced cells were selected with puromycin (1 µg/ml) for 72 h, and NRBP1 knockdown was induced by 1 µg/ml doxycycline for 48 h in MDA-MB-231 parental cells or 72 h in MFM-223 cells, or 10 ng/ml doxycycline for 48 h in MDA-MB-231_HM cells.

Introduction of GFP-Rac1_G12V and GFP-Cdc42_Q61L, or shRNA-resistant NRBP1 into MDA-MB-231 cells with NRBP1 knockdown was achieved by retroviral-mediated transduction. To generate retroviral particles, HEK-293T cells were co-transfected with VSVG, Gag-Pol and pMX-GFP-Rac1_G12V, pMX-GFP-Cdc42_Q61L or pMIG-NRBP1 using Lipofectamine 3000. After infection with retroviral particles for 24 h in the presence of polybrene (8 µg/ml), successfully transduced cells were sorted by flow cytometry on the basis of GFP expression levels.

### Transfections

Plasmid transfections were performed using Lipofectamine 3000 (Life Technologies, cat. L3000015) according to the manufacturer instructions. SMARTpool siRNA or individual siRNAs were obtained from Dharmacon and applied to cells using DharmaFECT1 or 3 (Dharmacon, Lafayette, CO) transfection reagent. Types of DharmaFECT reagents used in different TNBC cells were #1: MDA-MB-231 and #3: CAL-120 and MFM-223. NRBP1 siRNAs (M-005356-02-0005) sequences were #1: GAGAAGAGCAGAAGAAUCU, #2: GAACAGUACCCUCAACUCA, #3: GUGUAGAGGUUGUGUGGAA and #4: UGUGAAGACUUGUCGAGAA. P-Rex1 siRNAs (MQ-010063-01-0005) sequences were #1: GAGAUGAGCUGCCCUGUGA, #2: GAAAGAAGAGUGUACAAUC, #3: CCACGGACAUCAUGCGGAA, #4: AAGAUGGGACAGCGGAUUA. Negative control siRNA was ON-TARGET plus Non-targeting pool (Dharmacon, D-001810-10-20).

### Cell lysis, immunoprecipitation and pull-downs

Cell lysates for immunoblotting and immunoprecipitation (IP) were prepared using radioimmunoprecipitation (RIPA) buffer and normal lysis buffer (NLB), respectively [42]. To pull down GTP-Rac1/Cdc42, cells were cultured in starvation medium overnight. Cell lysates were prepared using Mg2+ Lysis/Wash Buffer (MLB, MERCK Millipore, cat. 20-168), and pre-cleared by incubating with glutathione agarose (GE Healthcare) at 4 ^o^C for 10 min with end-to-end rotation. For pulldown of GTP-Rac1/Cdc42, cell lysates were incubated with PAK-1 PBD agarose beads (MERCK Millipore, cat. 14-325) for an hour at 4 ^o^C with end-to-end rotation. For IP of overexpressed Flag-tagged proteins, cell lysates were incubated with anti-FLAG M2 affinity-agarose beads (Sigma, cat. A2220) at 4 °C for 2–4 h with end-to-end rotation. Following extensive washing with appropriate ice-cold lysis buffer, the bead-bound complexes were eluted with SDS-PAGE loading buffer (SLB) prior to Western blotting analysis.

### Cell biological assays

MTS assays were undertaken as instructed by the manufacturer (Promega). Transwell migration and invasion assays were essentially as previously described [43]. Transwell migration chambers with 8-μm pores were purchased from Corning (cat. CLS3464-48EA) and MERCK Millipore (cat. MCEP24H48). Matrigel invasion chambers that were pre-coated with Matrigel were purchased from Corning (cat. 354483). Prior to seeding, cells were treated with mitomycin C at 10 µg/ml for 1 h to prevent cell division. Colony formation assays were as described [44].

### Dihydroethidium (DHE) assay

Cell-based assay buffer and DHE assay reagent were provided by the DHE assay kit (abcam, cat. ab236206). One day before performing the assay, MDA-MB-231 cells were plated in 96-well plates, and cultured in normal growth media overnight. On the next day, growth media was replaced by cell-based assay buffer, and then cells were cultured with ROS staining buffer for 1 h at 37 ^o^C protected from light. Following that, ROS staining buffer was replaced by cell-based assay buffer. Finally, fluorescence was measured with an excitation wavelength between 480-520 nm and an emission wavelength between 570–600 nm using a Pherastar (BMG LABTECH) plate reader.

### Mass spectrometry-based proteomic screen

A panel of 24 TNBC cell lines previously screened by MS-based tyrosine phosphorylation profiling [19, 20] was subject to whole proteome analysis as previously described [45].

MS-based proteomic analyses were undertaken on control and shRNA#1-mediated NRBP1 knockdown MDA-MB-231 cells. NRBP1 knockdown was induced by 1 µg/ml doxycycline for 48 h. These analyses were performed in triplicate on independent biological replicates according to previously-published protocols [21, 46].

Proteins with altered expression following NRBP1 knockdown were defined with cut-off values of *p*-value ≤ 0.05, and a ≥ 2-fold change in either direction. Corresponding genes were interrogated using the Enrichr pathway analysis platform (https://maayanlab.cloud/Enrichr/). The Top 10 pathway enrichments in the Reactome 2022 dataset were ranked according to adjusted p-value.

### BioID-MS/MS screen

MDA-MB-231_EcoR cells stably expressing mycBioID2-pBabe-puro or mycBioID2-pBabe-NRBP1 were plated in growth medium in 15 cm dishes. When cells reached 70% confluency, 50 µM biotin (Sigma, cat. B4501) was added to the medium and the cells incubated for 20 h at 37 ^o^C, after which protein were extracted using modified RIPA lysis buffer. Then the cell lysates were transferred to 15 ml conical tubes, followed by sonication for two sessions with 30 pulses. Finally, lysates were centrifuged and supernatants were collected. Protein concentrations were quantified by BCA assay. Protein samples were incubated with Streptavidin agarose beads (GE Healthcare) at 4 ^o^C for 22 h. Beads were then subject to extensive washing with modified RIPA buffer then 50 mM ammonium bicarbonate pH 8. For 100 µg peptides, 1 µg trypsin was added to samples, followed by incubation at 37 °C overnight with agitation. After overnight digestion, beads were pelleted and the supernatants transferred to a fresh Eppendorf (EP) tube. The beads were then rinsed with mass spec-grade H_2_O, and these rinses were combined with the original supernatant. Following that, the pooled fractions were centrifuged at 16,000 x *g* for 10 min and supernatants were transferred to a new EP tube and lyophilized in a speed-vac. Then samples were resuspended in 0.1% formic acid and the peptide concentration measured using a Nanodrop. Finally, the concentrations of all samples were normalized and analysed by Mass Spectrometry (MS; Q-Exactive Plus Hybrid Quadrupole-Orbitrap from Thermo Scientific) in the Monash Proteomics & Metabolomics Facility (MPMF). Data were analysed using MaxQuant to obtain protein identifications and their respective label-free quantification (LFQ) values using in-house standard parameters. Statistical analysis was performed using an in-house generated R script. A cutoff of the adjusted *p*-value of 0.05 (Benjamini-Hochberg method) along with a log2 fold change of 1 was applied to determine significantly enriched proteins.

### Xenografts

All procedures involving mice were conducted in accordance with National Health and Medical Research Council (NHMRC) regulations on the use and care of experimental animals and the study protocol approved by the Monash University Animal Ethics Committee.

Power calculations performed indicated group sizes of *n* = 10 per treatment group were required to detect a significant difference in tumour growth inhibition of 50% between treated and control (significance level: 0.05; power: 90%). Animals were housed in 6 cages with 5 animals in each cage. Each cage was randomly assigned to the experimental groups without considering any other variable. Mice (*n* = 30) were randomly divided into three equal groups, non-targeting control, shNRBP1#1 and shNRBP1#2 groups.

To avoid error, all animals were injected with the same cell solution. 2 × 10^5^ MDA-MB-231_HM cells stably transduced with Tet-on NRBP1 shRNA#1, shRNA#2 or empty vector were suspended in 20 μl PBS and injected into the fourth mammary fat pad of 6-week-old female BALB/c athymic nude mice purchased from Animal Resources Centre (Canning Vale, Australia). The whole process of fat pad injection was performed under sterile conditions. One day post-injection, doxycycline was administered in the food (600 mg doxycycline/kg) (Specialty Feeds, Australia) until the end of the experiment. Mice were monitored every day in the first week, then monitored and imaged every 2 to 3 days using an AMI-HTX imaging system. Investigators were not blinded to the experimental group information. At the end of the experiment, the animals were humanely killed by CO_2_ asphyxiation and cervical dislocation, and xenografts were collected and weighed. Results are presented as mean+/− SEM of tumour volume.

### Tail-vein injection

Power calculations performed indicated group sizes of *n* = 10 per treatment group were required to detect a significant difference in tumour metastasis inhibition of 50% between treated and control (significance level: 0.05; power: 90%). Animals were housed in 6 cages with 5 animals in each cage. Each cage was randomly assigned to the experimental groups without considering any other variable. Mice (*n* = 30) were randomly divided into three equal groups, non-targeting control, shNRBP1#1 and shNRBP1#2 groups.

Prior to tail-vein injection, doxycycline was administered in the food for 5 days, and cells used for injection were pre-treated with 10 ng/ml doxycycline for 48 h. To avoid error, all animals were injected with the same cell solution. 1 × 10^6^ doxycycline pre-treated MDA-MB-231_HM cells stably transduced with Tet-on NRBP1 shRNA#1, shRNA#2 or empty vector were suspended in 100 μl PBS and injected into one of the two lateral tail veins of 6-week-old female BALB/c athymic nude mice. Doxycycline was administered in the food until the end of the experiment. Mice were monitored every day in the first week, then monitored and imaged every 2 to 3 days using an AMI-HTX imaging system. Investigators were not blinded to the experimental group information. At the end of the experiment, the animals were humanely killed by CO_2_ asphyxiation and cervical dislocation. Results are presented as mean+/− SEM of tumour volume.

### Immunohistochemistry (IHC)

Tumours were excised from BALB/c athymic nude mice at the experimental endpoint and fixed in 10% (v/v) buffered formalin. Fixed-tumours were then paraffin-embedded and sectioned at 4 µm onto Superfrost Plus slides. Immunohistochemistry was carried out using the DAKO Autostainer Link 48. Sections underwent dewaxing, heat-induced antigen retrieval using DAKO Target Retrieval Solution (S1699) at 98 °C for 30 min, endogenous peroxidases were quenched by applying Dako Real Peroxidase Blocking solution (S2023) for 10 min, followed by Dako Serum Free Protein Block (X0909) for 30 min. Then, primary antibody incubation using NRBP1 or Ki-67 antibody was followed by the Dako Envision + System – HRP Labelled secondary antibody incubation system. Subsequently, sections were counterstained with Dako Automation Hematoxylin Histological Staining Reagent (S3301). For the analysis, 10 random fields of vision images per sample were taken with ImageScope viewer and quantified using the Fiji ImageJ software (Version 1.52).

### Data mining

For survival analysis, mRNA expression and associated survival data from 1084 breast cancer patients as part of the TCGA dataset were downloaded from the cBioPortal for Cancer Genomics portal (https://www.cbioportal.org/). TNBC patient samples (number = 171) were extracted from the TCGA cohort and divided into three groups having either low, medium, or high expression of NRBP1 based on the 30% quantile. Survival analyses comparing overall survival and disease-free survival between the low and high groups were subsequently performed using the R package ‘survival’ (with *p* < 0.05 considered significant). Box and Whisker plots were generated using BC Gene-Expression Miner v4.8 (https://bcgenex.ico.unicancer.fr/). Analysis was conducted using the ‘expression’ tool using data obtained from the METABRIC dataset (*n* = 1980). Data were stratified by PAM50 subtypes. Welch’s test was used to determine global significance across all groups and Dunnett-Tukey-Kramer test was used between groups. cBioportal was used for gene alteration and expression analysis using the METABRIC dataset stratified by PAM50 + Claudin-low subtypes.

The Depmap platform (https://score.depmap.sanger.ac.uk) was used to determine the dependency on NRBP1 in breast cancer cell lines.

To characterize PREX1 mRNA expression across breast cancer cell lines, cell lines were first classified into basal and luminal subtypes as previously described [47]. Corresponding gene expression data were then extracted from the Cancer Cell Line Encyclopedia (CCLE) and compared by student’s t-test.

### Statistical analyses

Experimental data were subject to appropriate statistical analyses as detailed in the corresponding figure legends. All (IP/)Westerns and biological assays were undertaken in triplicate on independent biological replicates.

## Supplementary information

Supplementary Figures

Supplementary tables

## Data Availability

The MS proteomics data have been deposited to the ProteomeXchange Consortium through the PRIDE partner repository. The data generated in this study are available upon request from the corresponding author. The mass spectrometry proteomics data have been deposited to the ProteomeXchange Consortium via the PRIDE [48] partner repository with the dataset identifier PXD032168. The data generated in this study are available upon request from the corresponding author.
