# Supplementary material for: The pseudokinase NRBP1 activates Rac1/Cdc42 via P-Rex1 to drive oncogenic signalling in triple-negative breast cancer
Source: Oncogene. 2023 Jan 24;42(11):833–47. doi: 10.1038/s41388-023-02594-w (PMC10005955; doi:10.1038/s41388-023-02594-w)
Supplement: Supplementary file 1 — Supplementary Figures [file 41388_2023_2594_MOESM1_ESM.docx]

**Supplementary Information**

**The pseudokinase NRBP1 activates Rac1/Cdc42 via P-Rex1 to drive oncogenic signalling in triple negative breast cancer**

Xue Yang, Miguel Cruz, Elizabeth V. Nguyen, Cheng Huang, Ralf B. Schittenhelm, Jennii Luu, Karla J. Cowley, Sung-Young Shin, Lan K. Nguyen, Terry C C Lim Kam Sian, Kimberley C. Clark, Kaylene J. Simpson, Xiuquan Ma and Roger J Daly.


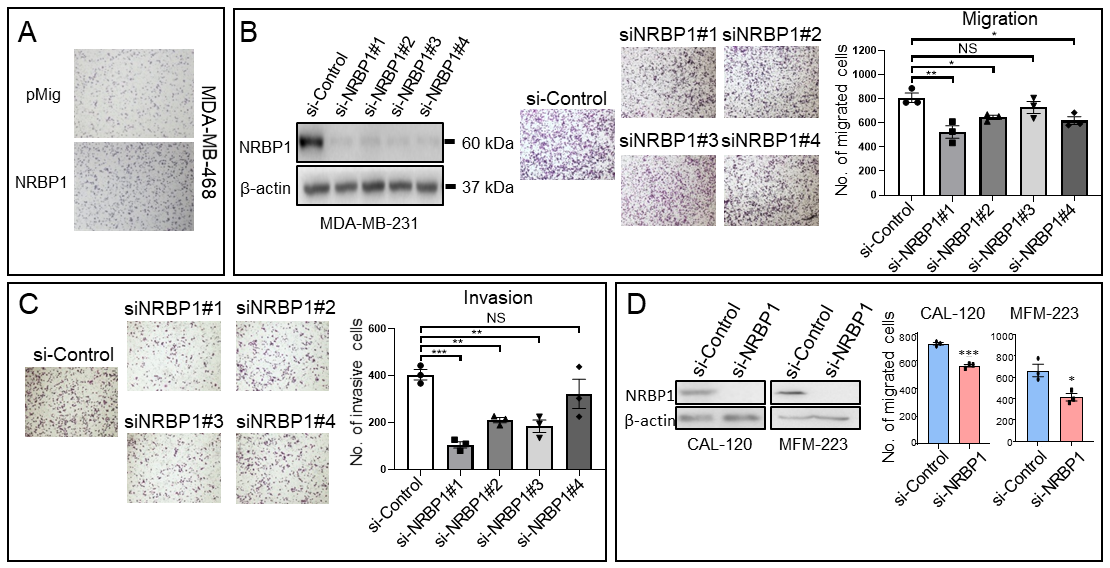


**Supp Figure 1. Effect of NRBP1 modulation on TNBC cell migration and invasion.** **A. Overexpression of NRBP1 enhances MDA-MB-468 cell migration.** Representative images of migration transwell assay. Quantification results are shown in Figure 2A. **B-C. Knocking down NRBP1 using deconvoluted siRNA pool reduces cell migration and invasion in MDA-MB-231 cells.** NRBP1 was knocked down using four individual siRNAs, with successful knockdown confirmed by Western blotting. Cells were subjected to migration and invasion transwell assays (B-C). Left panels, representative images of transwell assays. Right panels, quantification of transwell assays. **D. NRBP1 knockdown reduces migration in other TNBC cell lines.** Western blotting to confirm knockdown in each cell line and data from corresponding transwell migration assays are shown. Error bars represent the standard error of the mean from n=3 independent experiments. NS indicates p > 0.05, * = p < 0.05, ** = p < 0.01, *** = p < 0.001 by one-way ANOVA with Dunnett’s multiple comparisons test (B-C), or student’s t-test (D).


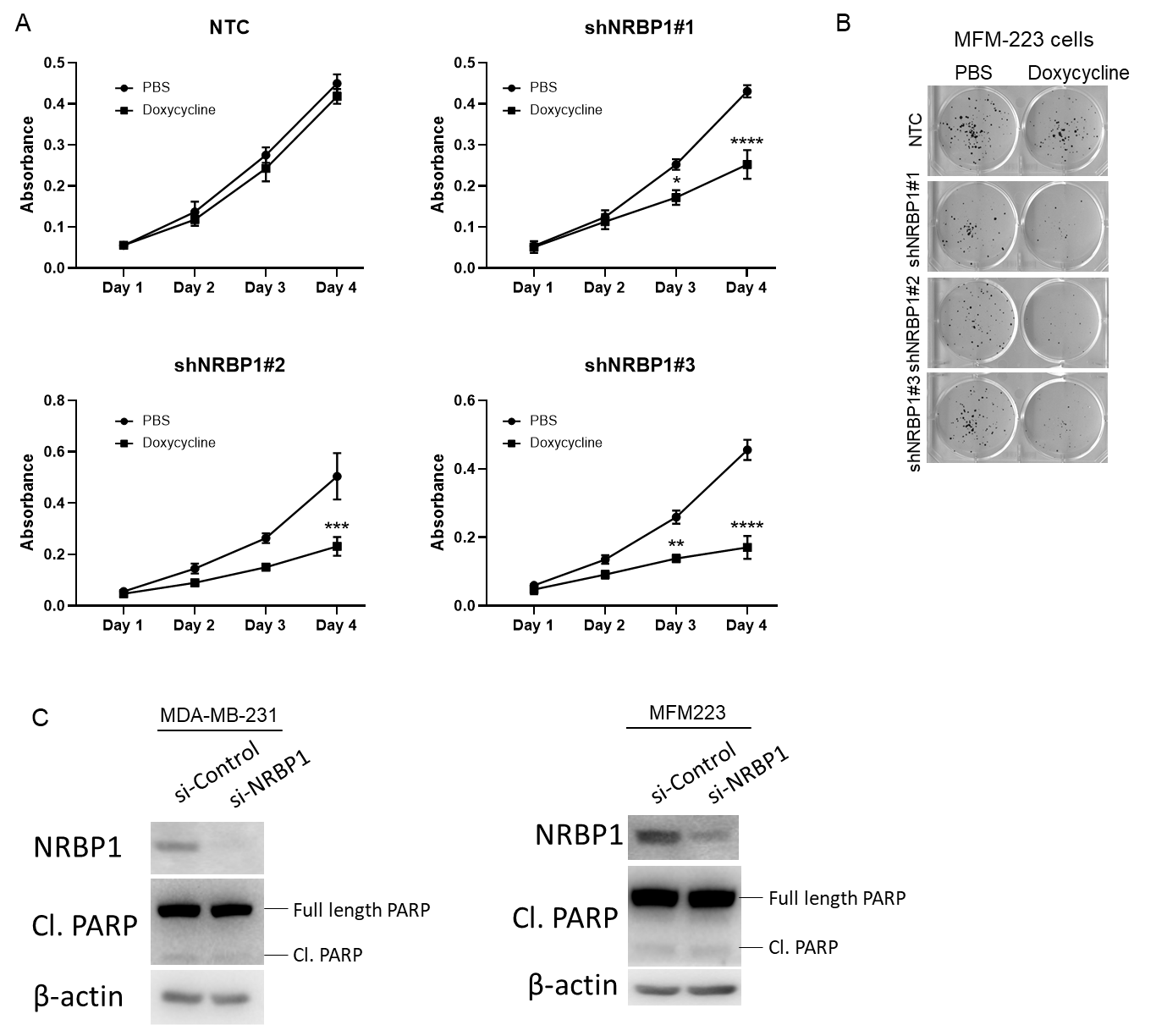


**Supp Figure 2. Effect of NRBP1 knockdown on cell proliferation. A. NRBP1 knockdown reduces monolayer proliferation in MDA-MB-231_HM cells.** Vector control (NTC) and inducible NRBP1 knockdown MDA-MB-231_HM cells were cultured +/- doxycycline and cell proliferation was determined by MTS assay. Error bars represent the standard error of the mean from n=3 independent experiments. * = p < 0.05, ** = p < 0.01, *** = p < 0.001, **** = p < 0.0001 by two-way ANOVA with Sidak’s multiple comparisons test. **B.** **NRBP1 knockdown reduces colony formation by MFM-223 cells.** Representative images of plates at Day 23. Quantification results are shown in Figure 3C. **C. Effect of NRBP1 knockdown on apoptosis in TNBC cell lines.** NRBP1 was knocked down using a siRNA pool in MDA-MB-231 (Left panel) and MFM-223 cells (Right panel). Total cell lysates were Western blotted for cleaved PARP.


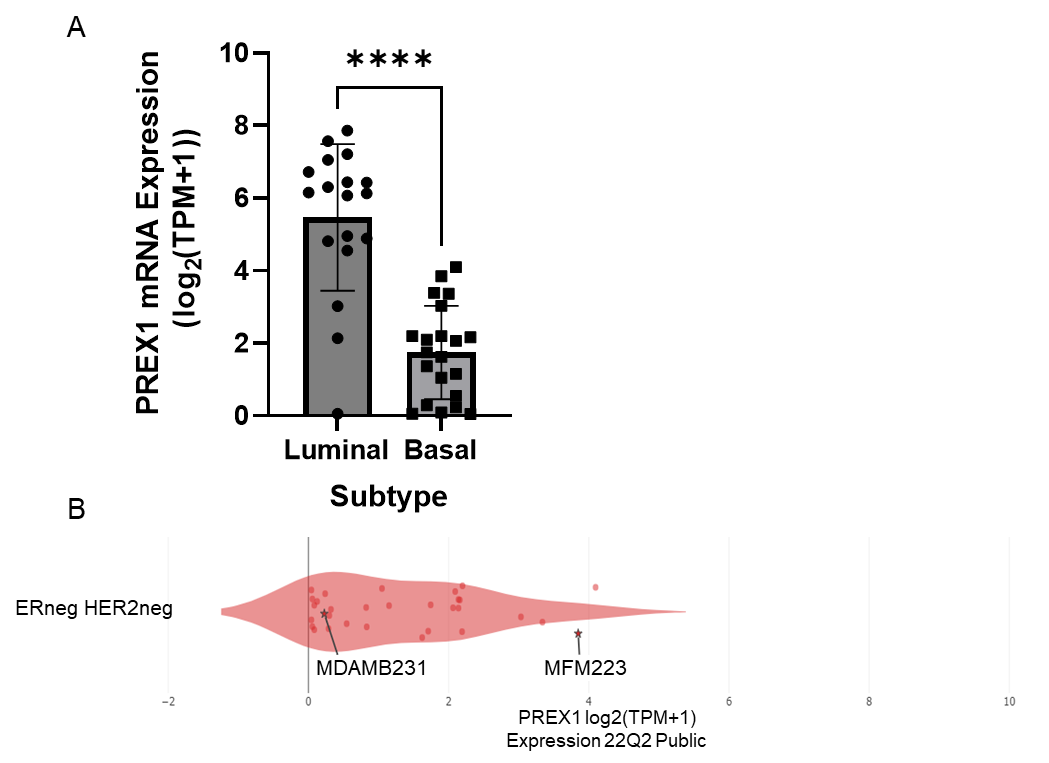


**Supp Figure 3. Characterization of P-Rex1 expression in luminal and basal breast cancer cell lines. A. P-Rex1 mRNA expression in basal and luminal breast cancer cell lines.** Cell lines were classified into basal (n=21) and luminal (n=18) subtypes based on classifications by Marcotte *et al*^28^. Gene expression data were extracted from the Cancer Cell Line Encyclopedia (CCLE). TPM = transcripts per million. Error bars represent the standard error of the mean. **** = p<0.001 by student’s t-test. **B. P-Rex1 mRNA expression in ER-negative HER2-negative breast cancer cell lines.** Data were extracted from the CCLE database and presented as a violin plot.


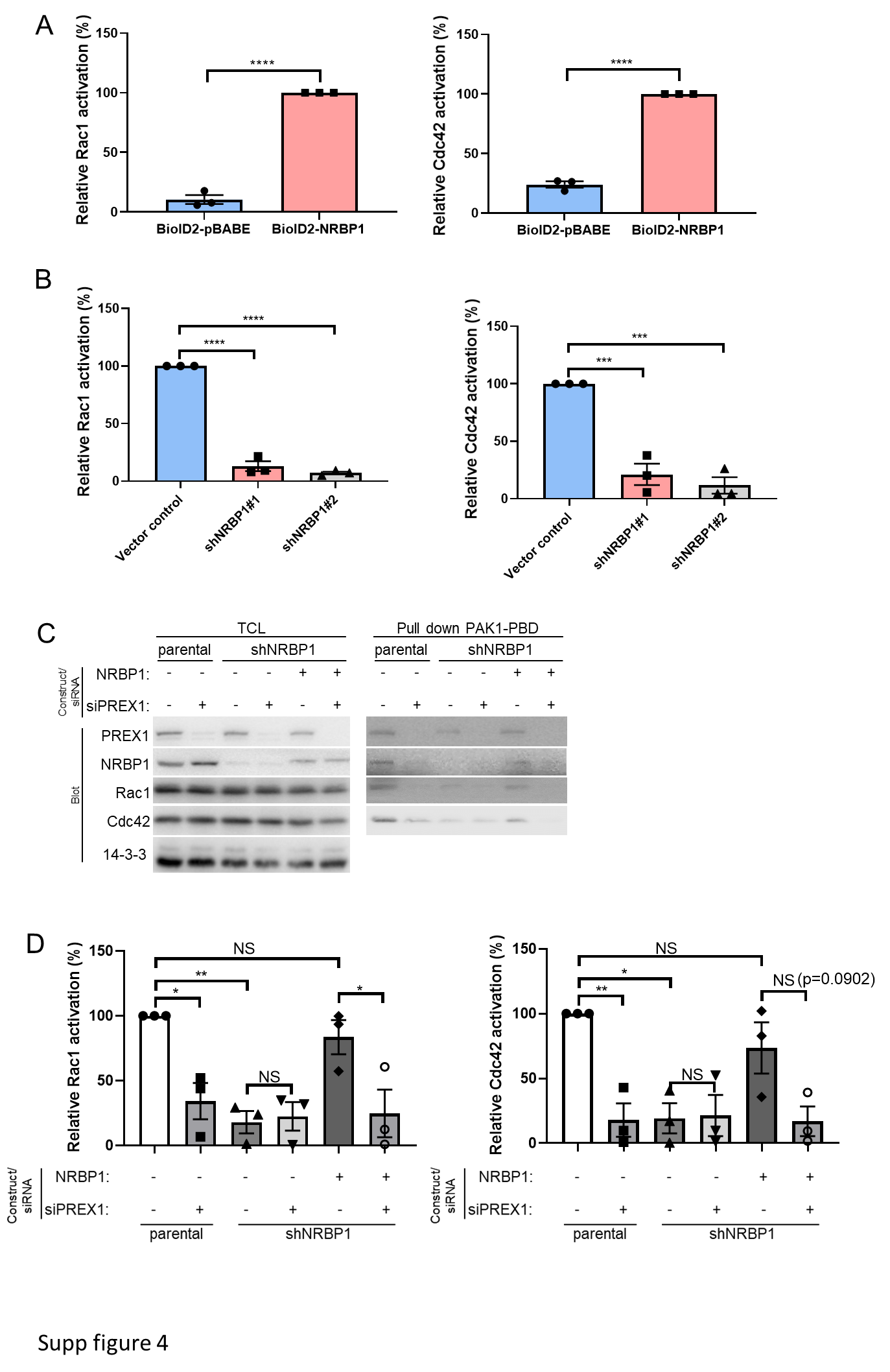


**Supp Figure 4. NRBP1 regulates Rac1/Cdc42 activation in TNBC via P-Rex1. A. NRBP1 overexpression enhances activation of Rac1 and Cdc42.** Densitometry results of Rac1/Cdc42 activation in MDA-MB-231_EcoR cells expressing mycBioID2-NRBP1 or the empty vector. Representative blots are shown in Figure 6A. Data are representative of n=3 independent experiments. **B. Stable knockdown of NRBP1 reduces the activation of Rac1 and Cdc42.** Densitometry results of Rac1/Cdc42 activation in MDA-MB-231_HM cells with shRNA-mediated NRBP1 knockdown or vector control. Representative blots are shown in Figure 6B. Data are representative of n=3 independent experiments. **C-D. NRBP1-mediated activation of Rac1 and Cdc42 is P-Rex1-dependent in MFM-223 cells.** MFM-223-shNRBP1 cells were complemented with shRNA-resistant NRBP1 in the presence or absence of siRNA-mediated P-Rex1 knockdown. Parental cells +/- P-Rex1 knockdown were also included as a control. Cell lysates and PAK-1 PBD pulldown assays were then Western blotted as indicated. Representative blots are shown in C, and densitometry results of Rac1/Cdc42 activation are shown in D. Data are representative of n=3 independent experiments. Rac1/Cdc42 activation was normalized to total Rac1/Cdc42, which was normalized to 14-3-3. Data are expressed relative to MDA-MB-231_EcoR cells expressing BioID-NRBP1 (A), MDA-MB-231_HM cells with vector control (B), or MFM-223 parental cells with si-Control (D), which were arbitrarily set at 100%. Error bars represent the standard error of the mean from n=3 independent experiments. NS indicates p > 0.05, * = p < 0.05, ** = p < 0.01, *** = p < 0.001, **** = p < 0.0001 by student’s t-test (A), one-way ANOVA with Dunnett’s multiple comparisons test (B), or two-way ANOVA with Tukey’s multiple comparisons test (D).


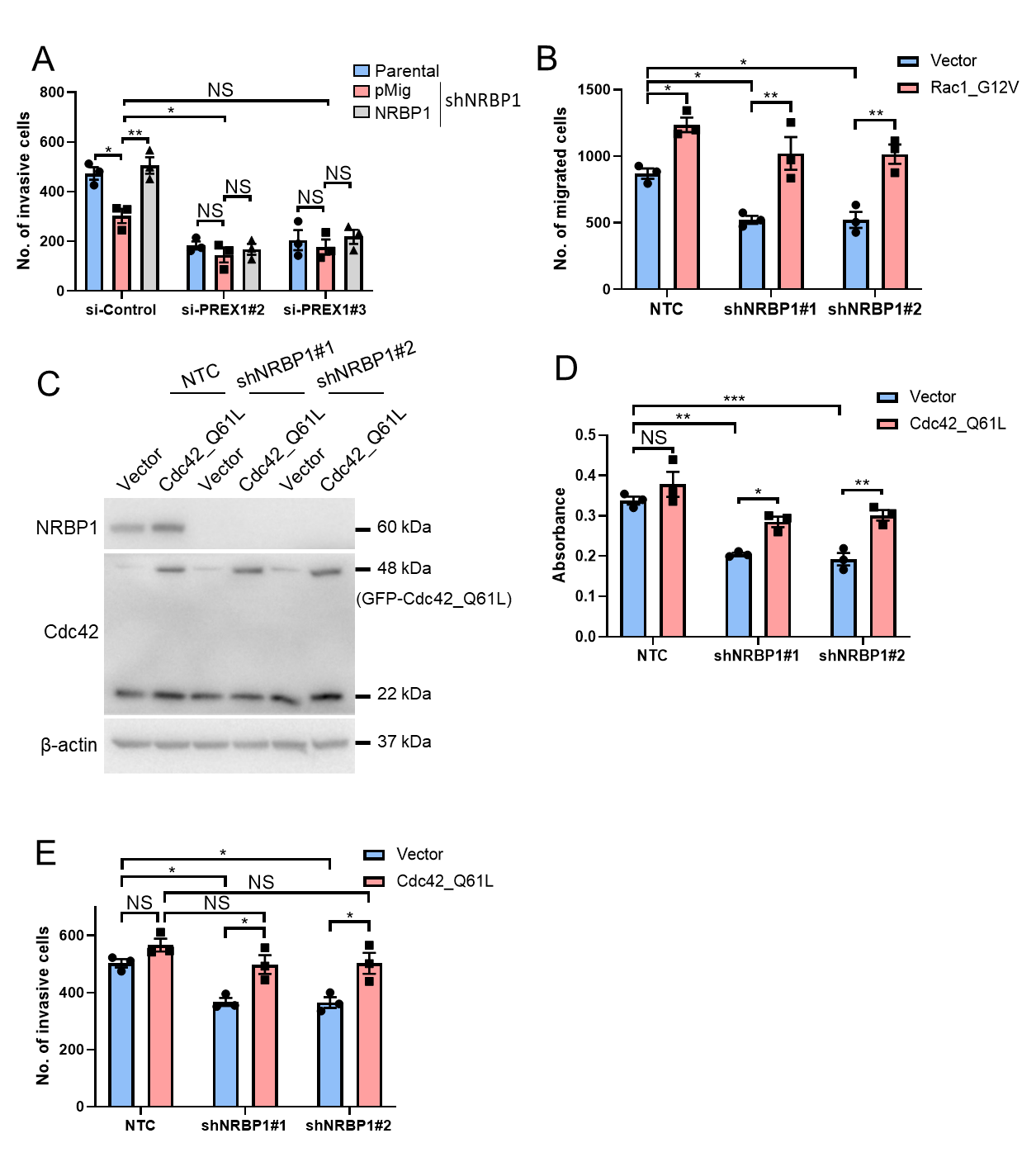


**Supp Figure 5. The P-Rex1/Rac1/Cdc42 axis plays essential roles in cellular functions and ROS regulation by NRBP1. A. Rescue of cell invasion by NRBP1 is P-Rex1 dependent.** Cells validated in Figure 7A were subjected to transwell invasion assays. **B. Effect of active Rac1 on migration of NRBP1-depleted cells.** Cells validated in Figure 7C were subjected to transwell migration assays. **C. Expression of Cdc42_Q61L.** MDA-MB-231 cells with shRNA-mediated NRBP1 knockdown were programmed to express GFP-Cdc42_Q61L. Cell lysates were Western blotted as indicated. **D. Effect of active Cdc42 on monolayer proliferation of NRBP1-depleted cells.** Cells validated in (C) were subjected to MTS assay. Data were obtained at Day 4. **E. Effect of active Cdc42 on invasion of NRBP1-depleted cells.** Cells validated in (C) were subjected to transwell invasion assays. Error bars represent the standard error of the mean from n=3 independent experiments. NS indicates p > 0.05, * = p < 0.05, ** = p < 0.01, *** = p < 0.001 by two-way ANOVA with Tukey’s multiple comparisons test.


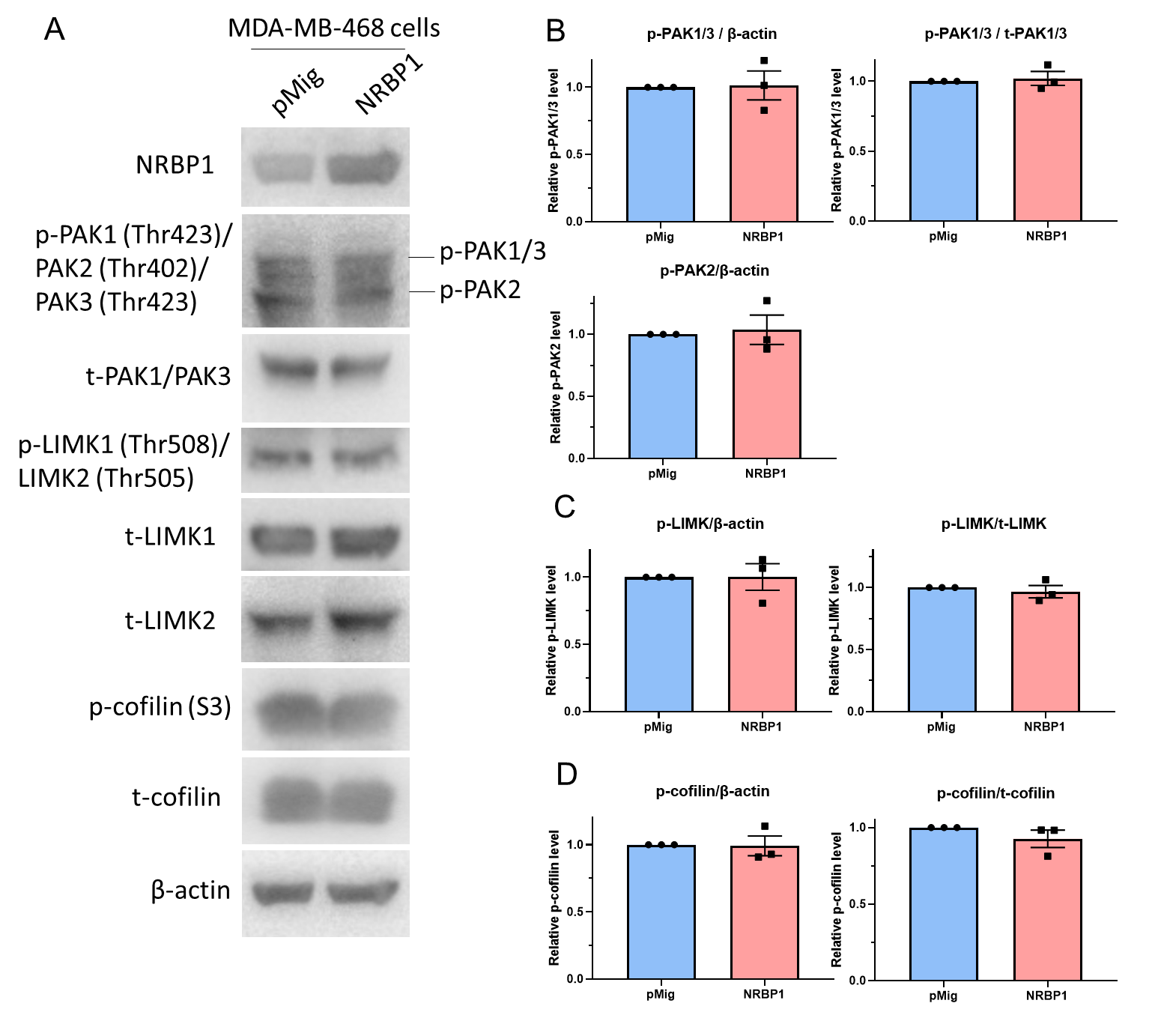


**Supp Figure 6. Effect of NRBP1 on Rac1/Cdc42 downstream effectors in MDA-MB-468 cells. A. Western blot analysis.** Lysates from cells transfected with an NRBP1 expression vector or empty vector (pMig) were blotted as indicated. **B-D. Densitometry results.** Data for phosphorylated PAK (B), LIMK (C), cofilin (D) normalized to either total protein or β-actin are presented. Error bars represent the standard error of the mean from n=3 independent experiments. Data were normalized relative to the pMig control that was arbitrarily set at 1.0. Total PAK2 was not detectable, so the phosphorylated PAK2 was only normalized to β-actin.


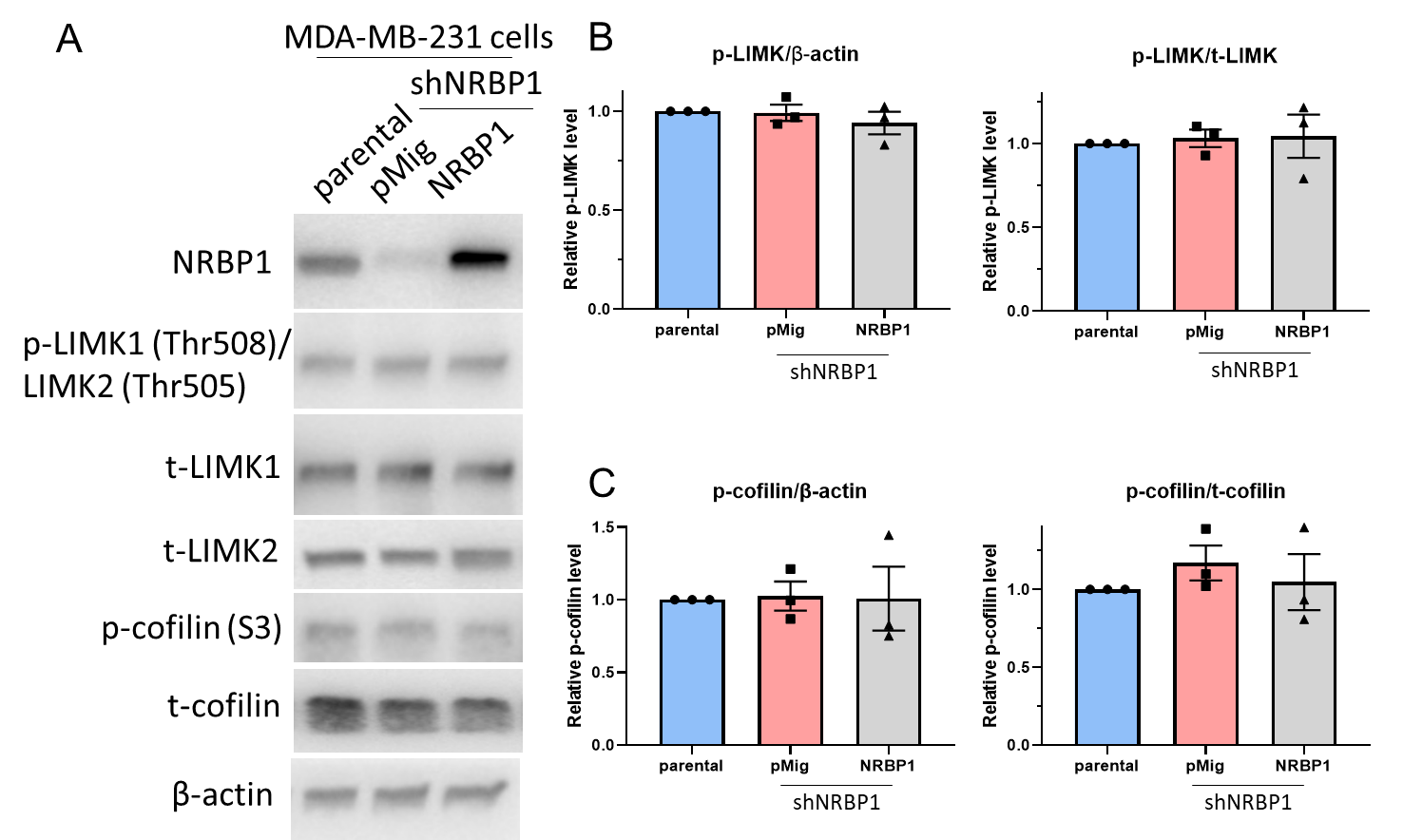


**Supp Figure 7. Effect of NRBP1 on Rac1/Cdc42 downstream effectors in MDA-MB-231 cells. A. Western blot analysis.** Lysates from MDA-MB-231 cells with shRNA-mediated NRBP1 knockdown or cells complemented with expression of shRNA-resistant NRBP1were blotted as indicated. Parental cells were included as a control. **B-C. Densitometry results.** Data for phosphorylated LIMK (B) and cofilin (C) normalized to either total protein or β-actin are presented. Error bars represent the standard error of the mean from n=3 independent experiments. Data were normalized relative to the parental cells that were arbitrarily set at 1.0.

**
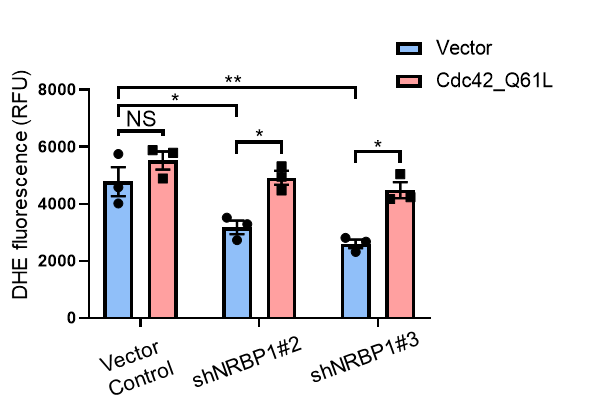
**

**Supp Figure 8: Roles of NRBP1 and Cdc42 in regulating ROS in MDA-MB-231 cells.** MDA-MB-231 cells with stable NRBP1 knockdown were programmed to express GFP-Cdc42_Q61L (Supp Figure 5C) and cellular ROS was assayed. Data are quantified using relative fluorescence units (RFU). Error bars represent the standard error of the mean from n=3 independent experiments. NS indicates p > 0.05, * = p < 0.05, ** = p < 0.01 by two-way ANOVA with Tukey’s multiple comparisons test.

**Supplementary Table 1. Results from BioID screen.** A list of 41 proteins identified as top-ranked NRBP1 interactors, with cutoff of adjusted p-value ≤ 0.05 and log2 fold change ≥ 1. Data are generated from n=3 independent experiments.

**Supplementary Table 2. Results for MS-based global proteomic analysis of NRBP1 knockdown cells.** Data are generated from n=3 independent experiments. Proteins with cut-off values of p-value ≤ 0.05 and a ≥ 2-fold change in either direction are indicated by shading.

**Supplementary Table 3. Pathway enrichment analysis of significantly altered proteins following NRBP1 knockdown.** A list of 56 genes identified as altered following knockdown according to the criteria in Supp Table 2 were submitted to Enrichr pathway analysis. Pathway enrichment was conducted using the Reactome 2022 dataset and ranked according to adjusted p-value.
